# Effect of Compositional and Processing Variations in New 5182-Type AlMgMn Alloys on Mechanical Properties and Deformation Surface Quality

**DOI:** 10.3390/ma12101645

**Published:** 2019-05-20

**Authors:** Paul Ebenberger, Peter J. Uggowitzer, Bodo Gerold, Stefan Pogatscher

**Affiliations:** 1 Chair of Nonferrous Metallurgy, Department Metallurgy, Montanuniversitaet Leoben, A-8700 Leoben, Austria; paul.ebenberger@stud.unileoben.ac.at (P.E.); peter.uggowitzer@mat.ethz.ch (P.J.U.); 2 Christian Doppler Laboratory for Advanced Aluminium Alloys, Montanuniversitaet Leoben, A-8700 Leoben, Austria; 3 Laboratory of Metal Physics and Technology, Department of Materials, ETH, CH-8093 Zurich, Switzerland; 4 AMAG Rolling GmbH, A-5282 Ranshofen, Austria; bodo.gerold@amag.at

**Keywords:** aluminium alloys, AlMgMn alloys, Lüders elongation, stretcher strain marks, quenching, particles

## Abstract

Laboratory-scale sheets of 5182-type AlMgMn alloys with varying Mg and Mn contents and additions of different amounts of Zn, Cu, Zr and Er were studied. The sheets were produced using two different cold-rolling degrees and two soft-annealing treatment procedures: air-circulated furnace annealing at 370 °C with subsequent furnace cooling, and salt-bath annealing with subsequent water quenching. Mechanical properties and deformation surface quality were studied via tensile tests with simultaneous visual surface characterization. The influence of the chemical composition and the processing route on grain size, mechanical properties, and surface quality is discussed in the study. A reduction in the Mg content improves the surface quality after plastic deformation, but at the expense of the mechanical properties. The results suggest the presence of an optimum Mn content in terms of optical appearance and mechanical properties. Adding Zr largely inhibits recrystallization, which is reflected in a textured microstructure. Adding Er affects neither the surface quality nor the mechanical properties. Specific combinations of Zn or Cu addition, cold-rolling degree, and heat treatment generate significant improvements in the mechanical and optical properties. In general, annealing at high temperature with subsequent quenching leads to enhanced surface quality and mechanical properties, and adding Zn enables further noteworthy improvements.

## 1. Introduction

AlMgMn alloys have a wide field of application, ranging from beverage cans to structural components [1]. Because of their well-balanced properties and favourable production costs, 5xxx series alloys have become a field of special interest in the automotive industry, complementing or even replacing 6xxx series alloys. The major drawback to using AlMgMn alloys in visible car parts, e.g., fenders or bonnets, is their non-decorative surface effects (Lüders lines and stretcher strain marks (SSM) type B), which occur during deforming at room temperature [1]. Investigations into the influencing factors and underlying mechanisms have been conducted for some time to understand, reduce or eliminate the Lüders elongation and dynamic strain ageing (DSA), which cause these various kinds of SSM.

Over time, different models have been developed to describe the mechanism behind Lüders elongation. The first concept involves the pinning of dislocations by Cottrell-like clouds of Mg atoms and subsequent breakaway, similar to carbon steel [2,3]. This hypothesis was later revised and replaced by more advanced concepts [4,5,6,7,8]. The current prevailing theory assumes that dislocations are fixed to grain boundaries that are decorated with Mg atoms. After reaching a critical stress dislocation, sources are activated and an avalanche of dislocations starts to move and pile up at the grain boundary. If the stress ahead of the pile-up is sufficient to operate the Mg-pinned dislocation segments of dislocation sources in the next grain or in the grain boundary, slip transfer takes place and continues from grain to grain.

While the correlation between SSM type B and DSA is undisputed, the explanation of the exact underlying mechanism has changed in the last few decades [4,5,6,7,8,9,10,11,12]. The established theory postulated the pinning of mobile dislocations by dislocation forests or aged dislocations and subsequent (pipe) diffusion of Mg atoms along the dislocation line [13,14]. Subsequent investigations showed that clusters of solute atoms at dislocation forests control the pinning behaviour [15,16]. Curtin et al. [17] proposed a mechanism based on single-atomic jumps of Mg atoms through the dislocation core. The consequent pinning of the dislocation causes the stress-strain behaviour during DSA.

**Figure 1:**
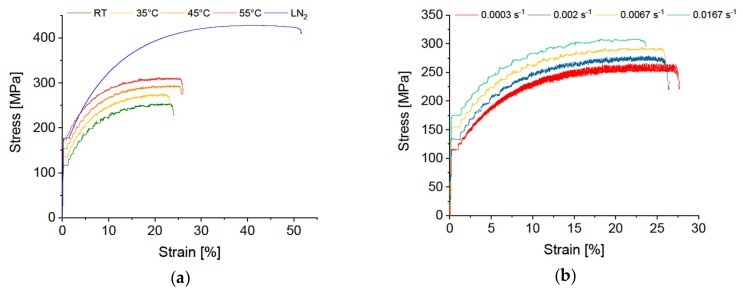
Stress-strain curves of commercial EN AW-5182 alloys depicting (**a**) temperature and (**b**) strain rate dependence of Lüders elongation and dynamic strain ageing. In (**a**) all curves except from 45 °C and LN_2_ (−196 °C) are shifted 15 MPa up or down. In (**b**) all curves except from 6.7 × 10^−3^ s^−1^ are shifted 20 MPa up or down.

In order to reduce or avoid Lüders elongation and DSA it is crucial to identify the influencing factors. While some measures, such as changing the deformation temperature [18,19,20,21,22] or the deformation rate [23,24,25] might influence the extent of DSA (see Figure 1), they are usually not applicable for the serial production of, e.g., outer car parts. Other feasible ways to minimize Lüders elongation and DSA and their optical manifestations are still needed.

Increasing grain size *d* is known to reduce SSM, in particular Lüdering [4,18,23,26,27,28,29,30,31,32,33,34,35]. Because of inconsistencies in the straightforward *d*^−0^^.^^5^ dependence of the Lüders elongation, the authors of this study recently proposed a new interpretation of this dependence [36]. It is well known that the processing route, including the final heat treatment and quenching in particular, influences Lüders elongation and DSA serrations [18,27,30,37,38,39,40]. The authors recently revealed that the size of primary constituents is largely responsible for the positive effect of the quenching procedure on the extent of the Lüders effect (Lüdering) [36].

Besides changing the thermomechanical production route, adapting the chemical composition is expected to contribute to improving the mechanical properties and surface quality of the sheets. Due to the underlying mechanisms, a reduction in Mg content might be the most plausible way to reduce SSM [27,28,30,35,41,42,43,44,45,46]. However, because a slightly reduced Mg content is unlikely to be sufficient to completely eliminate SSM, and adverse property changes might occur if the Mg content is too low, alloying with other elements such as Mn, Zn, Cu and Zr or a combination of them may have a positive impact [47,48,49,50,51,52,53,54,55,56,57,58]. Cu and Zn additions, in particular, have shown potential for improving mechanical properties as well as reducing SSM [47,48,49,50].

Although much research has been done over the years, it is hard to compare results because of significant differences between individual investigations. These involve different compositions, casting techniques and thermomechanical processing routes, to name only the main variables.

The aim of this study is to evaluate the influence of numerous alloying elements and different thermomechanical processing routes on the mechanical properties and visual appearance during and after the deformation of an AlMgMn alloy. For this purpose, laboratory batches were produced from EN AW-5182 with a standard chemical composition and alloys based on 5182 with varying Mg, Mn, Zn and Cu contents plus Zr and Er additions. Sheets were produced with final cold-rolling degrees of 63% and 20% and two different final heat treatments: (i) annealing in an air-circulated furnace with slow cooling and (ii) annealing in a salt bath with subsequent water quenching. A special focus was the compositional and processing effect on Lüders elongation and the accompanying Lüders lines.

## 2. Materials and Methods

For this study, 11 different alloys based on EN AW-5182 were produced from commercially pure elements (>99.9 wt %) and master alloys (Al-75 wt % Fe, Al-75 wt % Mn, Al-10 wt % Zr); the chemical compositions are shown in Table 1. The synthesis was performed in a resistance-heated furnace Nabertherm K20/13/S (Lilienthal, Germany), with a graphite crucible. Degassing of the melt was carried out with N_2_ using an impeller. The alloys were cast with a cooling rate of 1–2 K/s in laboratory-scale moulds and milled to a final block size of 174 × 84 × 44 mm^3^.

For the alloys with Cu, Zn, Zr and Er additions, homogenization treatments were carried out prior to the rolling process. The Zn-containing alloys were homogenized at 460 °C for 24 h, and the Cu-containing alloys were homogenized for 24 h at 480 °C and 24 h at 490 °C to avoid melting at the grain boundaries. To optimize the formation of Zr and Er dispersoids these two alloys were annealed according to a three-step scheme: 4 h at 350 °C, 24 h at 480 °C and 24 h at 490 °C [59,60,61].

The alloys were hot- and cold-rolled (CR) on a laboratory scale from 40 (slab) to 1.2 mm. The milled slabs were heated to the hot-rolling temperature of 530 °C. In the final rolling step the sheets were cold-rolled to achieve two different cold-rolling degrees: 63% and 20%, respectively (note: alloy Cu2 was 63% cold-rolled only).

**Figure 2:**
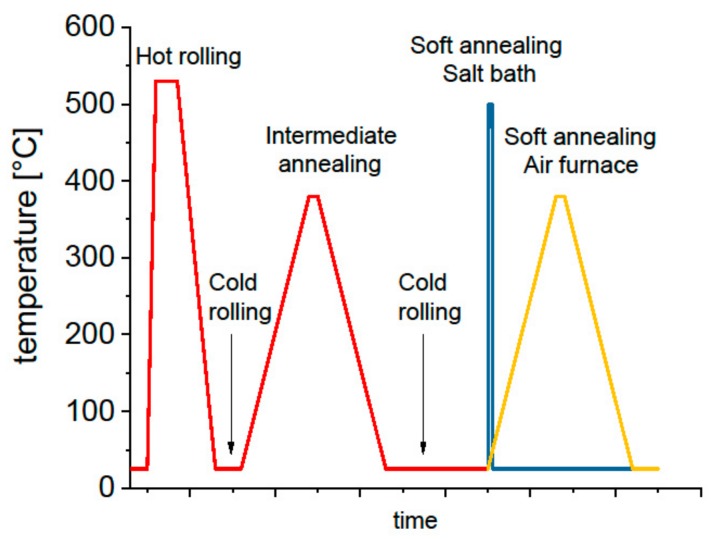
Schematic depiction of the thermomechanical processing route.

For all alloys the heat treatment procedure included intermediate annealing at 370 °C in an air-circulated furnace (ACF) prior to final cold rolling and two different terminal soft annealing treatments: in an air-circulated furnace at 370 °C for 1 h with subsequent furnace cooling and in a salt bath at 500 °C for 5 min with subsequent water quenching (SB), respectively. The thermomechanical processing route is depicted schematically in Figure 2. Moreover, the conditions together with the sample designations are summarized in Table A1 in Appendix A.

To evaluate the mechanical properties of the alloys, uniaxial tensile tests were carried out on a ZWICK-ROELL BT1-FR100THW (Ulm, Germany) universal testing machine with a 50 kN load cell on 80 mm gauge length specimens in accordance with EN-ISO 6892-1. The stiffness of the tensile test setup amounts to 8 kN/mm². The σ-ε curves shown represent an average out of three tested specimens.

The microstructure was investigated via light optical microscopy (LOM) Zeiss Axio Imager M1m (Jena, Germany) and scanning electron microscopy (SEM) JEOL 7200F FEG-SEM (Tokyo, Japan).

To rate the optical appearance of the deformation surface with regards to stretcher strain marks (Lüders lines and SSM type B), all tensile tests were visually inspected and the entire progress of formation and evolution of the surface markings was classified according to a five-step system, where grade 1 reflects neither visible Lüders lines nor SSM type B, and grade 5 severe surface markings throughout the deformation of the specimen. Note that this rating only applies to the tensile test specimens described above.

## 3. Results

The effect of compositional variations and process modifications on the different alloys is presented in the following sections. Changes in the plastic flow behaviour, the mechanical properties, the surface quality, and the microstructure are assessed individually.

### 3.1. Mechanical Properties

A complete dataset of the mechanical properties of all alloy variations is given in Table A2 in Appendix A.

#### 3.1.1. Standard 5182

**Figure 3:**
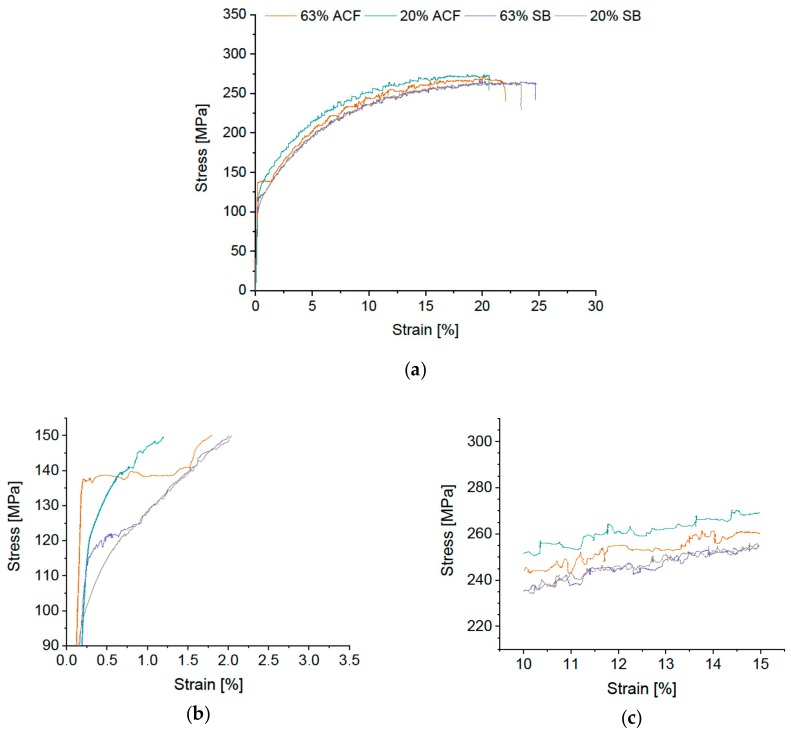
(**a**) Stress-strain diagram of standard EN AW-5182 with cold-rolling degrees of 63% and 20%, soft annealing in air-circulated furnace (ACF) at 370 °C and fast salt-bath (SB) heat treatment at 500 °C with subsequent water quenching. Details of (**b**) Lüders elongation and (**c**) the sector 10–15% elongation to depict stress serrations due to dynamic strain ageing.

Figure 3 depicts the stress-strain curves of standard 5182 in all four conditions. The data for the 63% cold-rolling degree and soft annealing in the air-circulated furnace (ACF) show a typical σ-ε curve of an AlMgMn alloy, with pronounced Lüders elongation ε_L_ and stress serrations due to DSA. In contrast, the salt bath (SB)-treated specimen shows just a kink in the curve but no distinct Lüders elongation. At the 20% cold-rolling degree either heat treatment generates complete or almost Lüdering-free stress-strain curves. Figure 3b,c shows the details.

The DSA serrations, and thus SSM type B, are reduced in both rapidly quenched SB conditions (Figure 3c).

Generally, a higher cold-rolling degree generates higher yield stress, independent of the heat treatment, and the strain hardening stays unchanged; see Figure 3b for details. Here and in the following, we do not comment on the influence of alloy modification on elongation values. However, the data on elongation to failure of all alloys are listed in Table A1.

#### 3.1.2. Solid-Solution Strengthening: Mg

**Figure 4:**
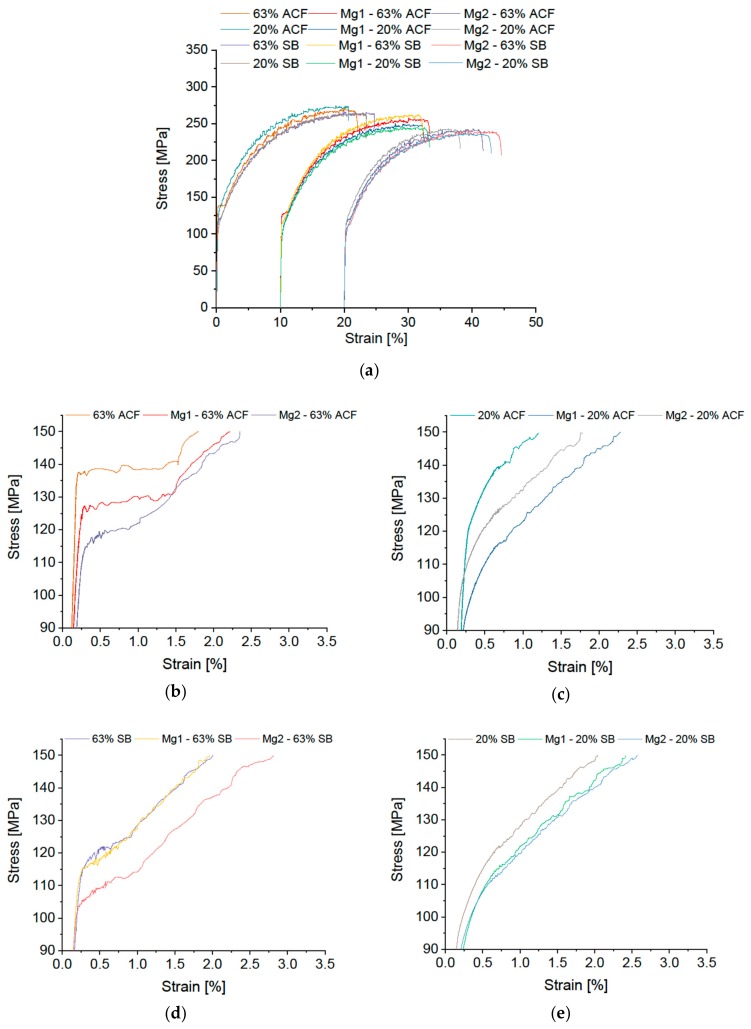
Stress-strain diagram of modified EN AW-5182 with differing Mg content (Standard 4.57%, Mg1 4.16%, Mg2 3.60%). (**a**) Details of Lüders elongation: soft annealing in ACF at 370 °C with (**b**) 63% and (**c**) 20% cold rolling; salt-bath treatment at 500 °C with subsequent water quenching with (**d**) 63% and (**e**) 20% cold rolling.

For the Mg variations with 4.16 wt % and 3.60 wt %, some distinct trends regarding the mechanical properties are worth noting; see Figure 4.

With 63% CR and furnace heat treatment, all alloys exhibit pronounced Lüders elongation, whereas with 20% CR almost no Lüdering is visible. The SB variants with 63% CR display a slight kink in the σ-ε curve, while with 20% CR no Lüders elongation is visible. Generally, the yield stress decreases with decreasing Mg content.

#### 3.1.3. Dispersoid Formers: Mn, Zr, Er

**Figure 5:**
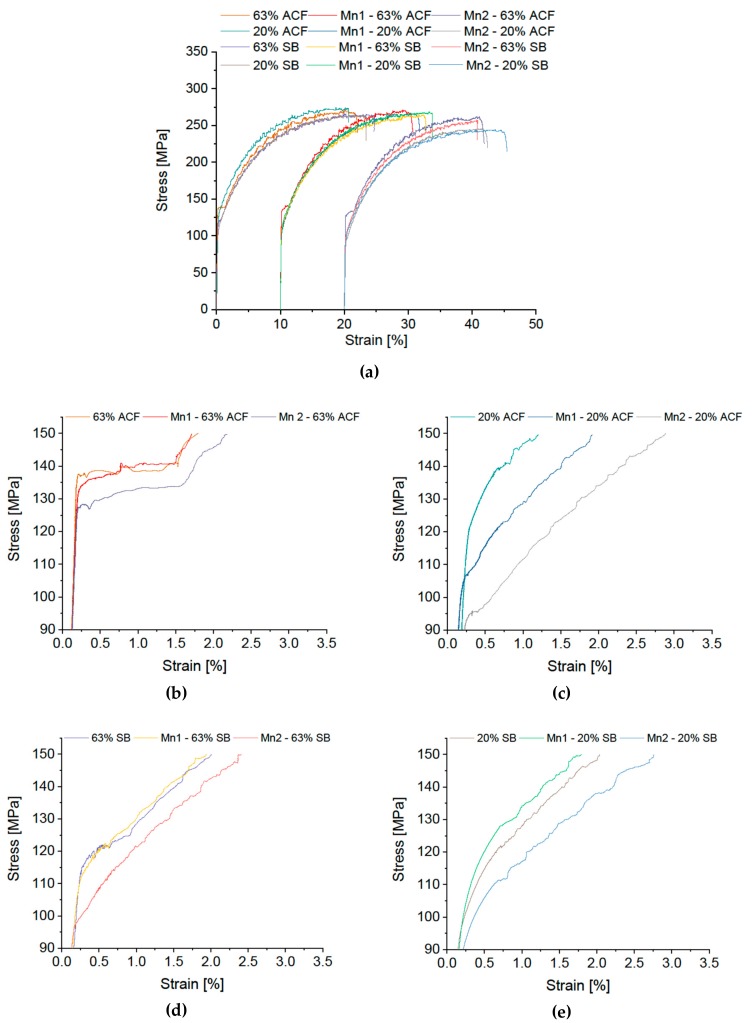
Stress-strain diagram of modified EN AW-5182 with differing Mn content. (**a**) Overview; Details of Lüders elongation: soft annealing in ACF at 370 °C with (**b**) 63% and (**c**) 20% cold rolling, salt-bath treatment at 500 °C with subsequent water quenching with (**d**) 63% and (**e**) 20% cold rolling.

**Figure 6:**
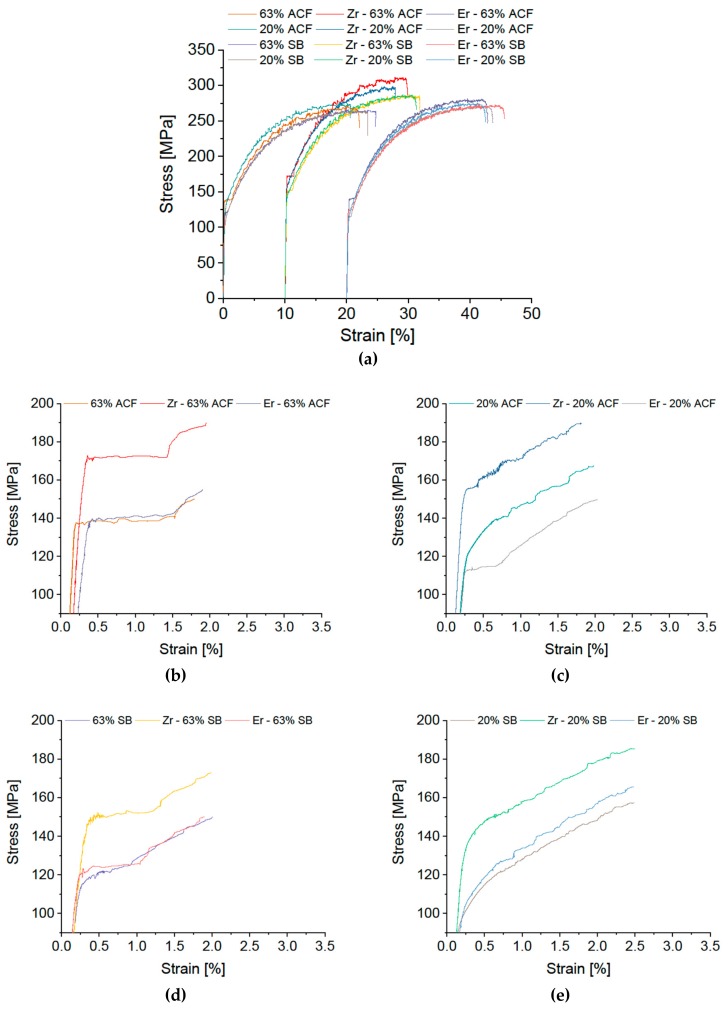
Stress-strain diagram of modified EN AW-5182 with additions of Zr and Er. (**a**) Overview; Details of Lüders elongation: soft annealing in ACF at 370 °C with (**b**) 63% and (**c**) 20% cold rolling, salt-bath treatment at 500 °C with subsequent water quenching with (**d**) 63% and (**e**) 20% cold rolling. Please note the different scale in (**b**–**e**).

This section describes the effects of Mn, Zr and Er content on mechanical properties. The corresponding σ-ε diagrams are shown in Figure 5 and Figure 6.

The standard alloy with 0.41 wt % Mn is compared with 0.34 wt % and 0.20 wt % Mn variants. The σ‒ε curves reveal pronounced Lüdering for 63% CR plus AFC for all Mn variations. For the alloys with higher Mn content a small kink in the σ-ε curves is visible for 63% CR plus SB, while the alloy with low Mn content exhibits a fairly smooth curve.

With 20% CR plus ACF a slight kink occurred in both Mn modifications, predominantly in the 0.2 wt % Mn alloy. For a low degree of cold rolling and SB, no Lüdering is evident. Regarding the DSA serrations, no influence of the Mn content is recognizable. The Mn-rich alloys also show comparable strength levels for all combinations of cold-rolling degree and heat treatment. The Mn-poor alloy, however, exhibits significantly lower strength.

The Zr- or Er-modified alloys show no improvement in Lüders or DSA serrations appearance compared to the standard alloy. On the contrary, a slight worsening with Zr or Er addition is visible in the variants 20% CR plus ACF and 63% CR plus SB. A clear increase in the yield stress is recognizable for the Zr variant, however. By contrast, Er has no significant effect on the mechanical properties.

#### 3.1.4. Precipitate Formers: Cu, Zn

**Figure 7:**
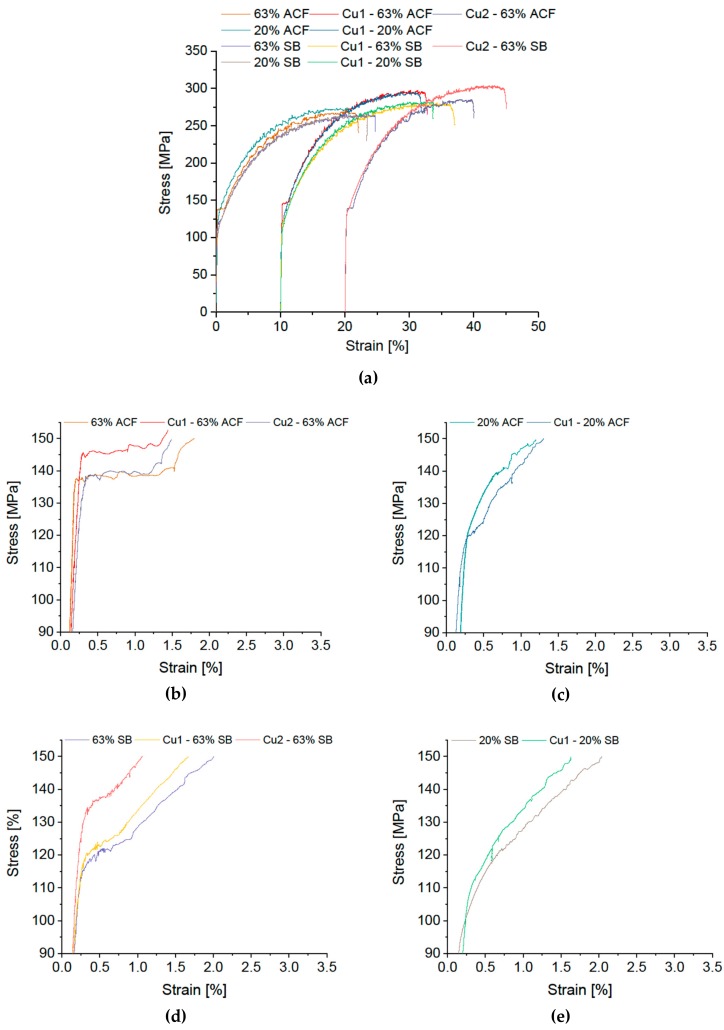
Stress-strain diagram of modified EN AW-5182 with differing Cu content. (**a**) Overview; Details of Lüders elongation: soft annealing in ACF at 370 °C with (**b**) 63% and (**c**) 20% cold rolling, salt-bath treatment at 500 °C with subsequent water quenching with (**d**) 63% and (**e**) 20% cold rolling.

**Figure 8:**
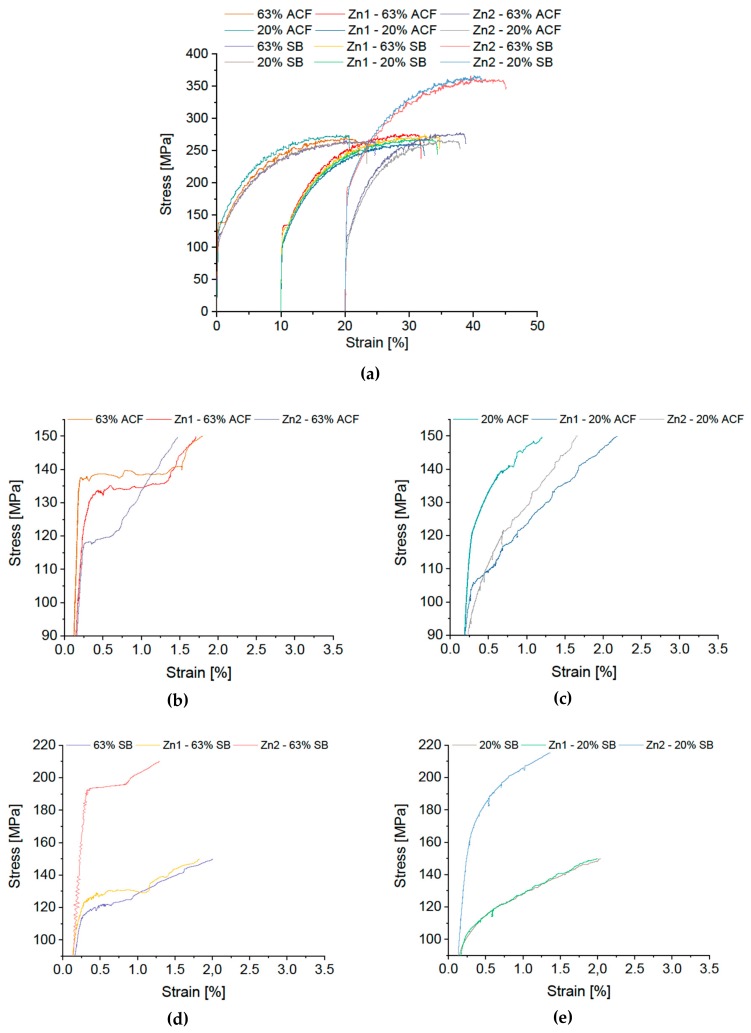
Stress-strain diagram of modified EN AW-5182 with differing Zn content. (**a**) Overview; Details of Lüders elongation: soft annealing in ACF at 370 °C with (**b**) 63% and (**c**) 20% cold rolling, salt-bath treatment at 500 °C with subsequent water quenching with (**d**) 63% and (**e**) 20% cold rolling. Please note the different scale in (**a**,**d**,**e**).

The effect of adding different amounts of copper (0.15 or 0.75 wt %) and zinc (0.24 and 2.08 wt %) is shown in Figure 7 and Figure 8 (note that the high-copper alloy Cu 2 was cold-rolled to a degree of 63% only).

The σ-ε curves in Figure 7 illustrate that alloying Cu has only a marginal influence on Lüders strain and on DSA serrations. The condition 63% CR plus ACF shows a pronounced Lüdering for all variants. In the condition 63% CR plus SB the Cu-modified alloys show a σ-ε kink, similar to the reference. Of interest, however, is the significant increase in the yield stress of the rapidly cooled SB variant with increasing Cu content.

As with the Cu modification, a more pleasing picture is obtained for the Zn variants. Although distinct Lüdering appears in all modifications in condition 63% CR plus ACF and noticeable kinks occur for 63% CR plus SB, the 20% CR conditions are more or less free of Lüders elongation. A particularly striking change resulting from Zn addition is seen in the yield stress. The Zn-rich variant also shows a significant increase of >80 MPa, but only where rapid-cooled SB treatment has been applied.

### 3.2. Surface Quality

Since the sheet surface is a critical quality feature in potential applications, we examined the sample surface throughout the deformation process. To provide a comparable classification of visual appearance, we evaluated the Lüders lines and the SSM type B on a five-point scale in tension tests, with a low rating number indicating better visual appearance. Table 2 summarizes the ratings for all alloys and conditions. The occurrence of a discontinuity in the σ-ε curve (kink) is listed as ε_L_ but marked with ‘k’. If there is an orange peel effect its extent is annotated. The occurrence of orange peel effect may overlap with Lüders lines. Where this occurs, the classification of Lüders lines is more difficult. In Table 2 we have marked the relevant cases with an asterisk.

In general, the ACF sheets show a more pronounced orange peel effect. This may mask any Lüders lines that appear. In comparison, salt-bath annealed samples with 20% CR show only weak signs of the undesirable orange peel effect.

Table 2 indicates that the occurrence of Lüdering in the form of a kink results in less visible or no Lüders lines.

The surface quality of the tested specimens can be classified into three categories:

- Lüders lines and type B stretcher strain marks, both pronounced: 63% CR plus ACF; minor deviations in the severity of the SSM type B for the different alloys.
- No or few Lüders lines, weak orange peel effect and reduced SSM type B: 20% CR plus ACF as well as SB; usually SB produces weaker SSM type B than ACF.
- No or almost no Lüders lines, no orange peel effect, reduced SSM type B: 63% CR plus SB; overall this combination provides the best surface quality averaged over all alloy modifications.

Certain combinations of alloy modifications and process parameters generated a particularly attractive surface appearance:

- The Mg-reduced alloy variants display a smooth surface with reduced stretcher strain marks. This advantage is particularly evident for 63% CR plus SB.
- The Mn-reduced alloys have a slightly better surface quality compared to the standard EN AW-5182 plus SB treatment.
- The 2 wt % Zn-containing alloy in SB condition displays a fairly good optical appearance. Interestingly, adding Zn reduces the extent of SSM type B serrations considerably in the plastic strain range up to 5% (in particular for the SB treatment).

### 3.3. Microstructure

#### 3.3.1. Grain Size and Shape

**Figure 9:**
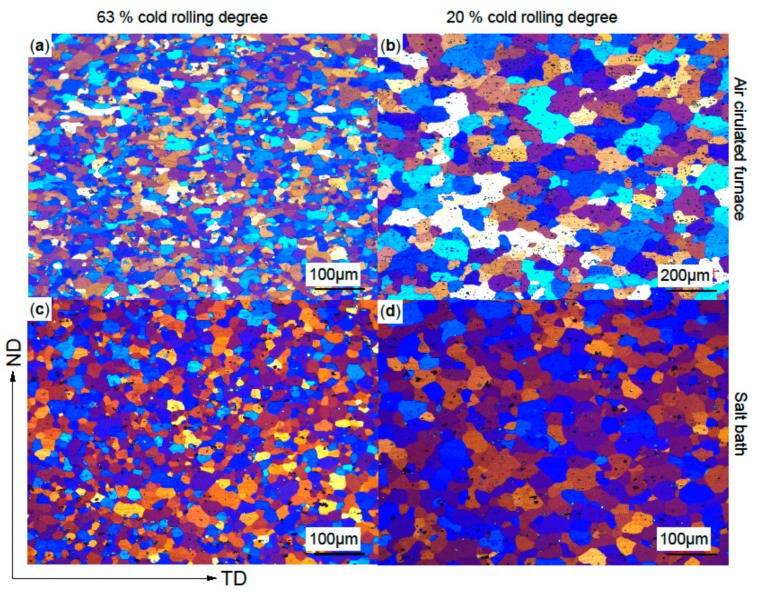
Light optical micrographs of typical microstructures for 63% (**a**,**c**) and 20% (**b**,**d**) cold rolling and soft annealing in an air-circulated furnace (**a**,**b**) and in a salt bath with subsequent water quenching (**c**,**d**). Please note the different scale for the air-circulated furnace heat treatment at 20% cold-rolling degree. Normal direction (ND), transversal direction (TD).

The various cold-rolling degrees, annealing and quenching procedures result in four characteristic microstructures. Illustrative light optical micrographs of these types are given in Figure 9. Exact data on the mean grain sizes are given in Table 2.

The procedure 63% CR plus ACF yields grains with uneven, jagged grain boundaries and broad grain size distribution, illustrated in Figure 9a. For all alloys the resulting grain size was in the range of 12‒18 µm, independent of alloy modification.

Sheets processed with 20% CR plus ACF show significantly enlarged grains (Figure 9b), with the exception of the Zr-modified alloy. In 63% and 20% CR the grain shape is similar, i.e., slightly compressed in the T direction.

The microstructure after SB treatment is different: the grains are spherical, with straight grain boundaries (see Figure 9c,d). The grain size variation is considerably reduced after SB treatment. At 63% CR the grain size is about 15 µm, except for Mn2 (0.2 wt % Mn) and Cu1 (0.15 wt % Cu), which have significantly larger grain sizes of ≈27 µm and ≈24 µm, respectively; 20% CR resulted in larger grains, between 35 and 40 µm for all alloy variations except the Zr variant.

**Figure A1:**
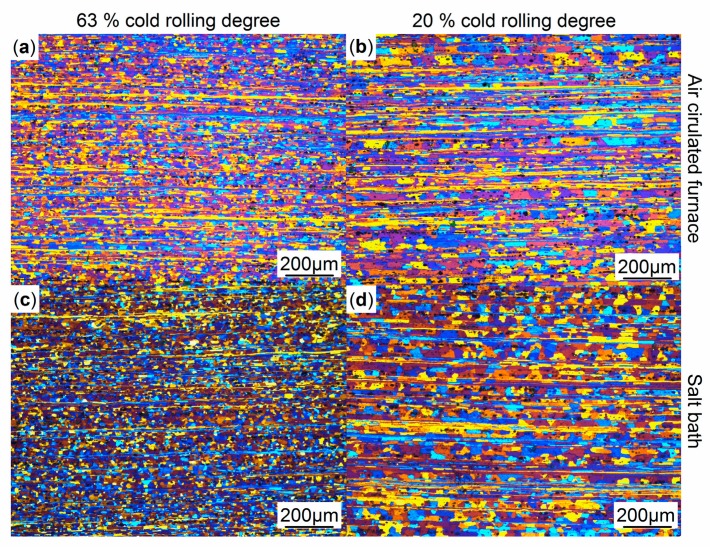
Light optical micrographs of the Zr-bearing alloy variation for 63% (left column; **a**,**c**) and 20% (right column, **b**,**d**) cold rolling and soft annealing in an air-circulated furnace (**a**,**b**) and in a salt bath with subsequent water quenching (**c**,**d**). Please note the different scale for the air-circulated furnace heat treatment at the 20% cold-rolling degree.

Interestingly, in SB condition adding 0.15 wt % Zr produces a partially non-recrystallized microstructure where the rolling structure is still apparent. Example light optical micrographs of the Zr-containing alloy are shown in Figure A1 in Appendix A. This does not apply to the Er-containing alloy modification.

#### 3.3.2. Constituents

**Figure 10:**
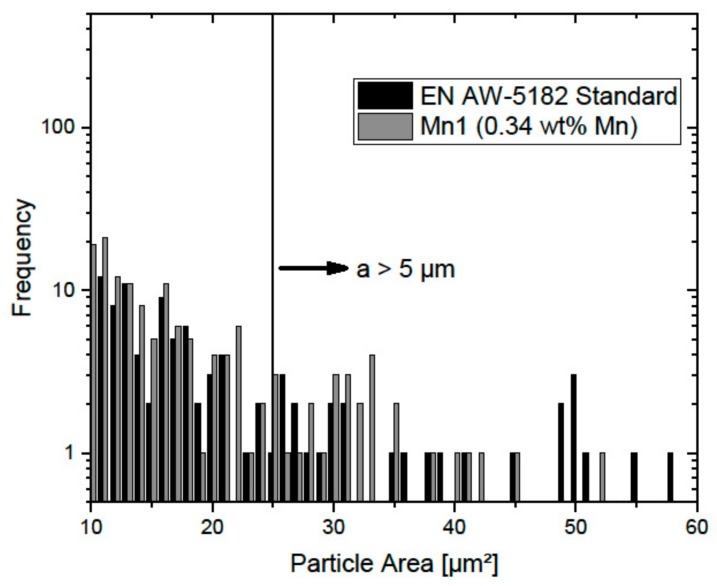
Histogram of particles with more than 10 µm² area for standard EN AW-5182 (0.41% Mn) and the Mn1 modification; note the relatively high fraction of constituents larger than 25 µm² (*a* > 5 µm).

The microstructure of the standard alloy AW-5182 usually contains constituents of type Al_13_(Mn,Fe)_6_ and Al_15_FeMn_3_Si_2_, which are formed during solidification. Their presence is important not only for grain size control during hot-rolling and heat treatment procedures, but also—as elucidated in the Section Discussion—for the creation of mobile dislocations. As an example, Figure 10 shows the histogram of the constituent distribution of the standard EN AW-5182 with 0.41% Mn and the Mn 1 alloy with 0.34% Mn. A considerable number of coarse (*a* > 5 μm) constituents can be found in both alloys, with a slight reduction for the reduced Mn content. 

## 4. Discussion

The experimental results indicate the following trends in the appearance of Lüders strain and DSA serrations, surface quality and yield stress:

(i) Independently of alloy modification, a lower final cold-rolling degree (20% CR) results in a reduction of the Lüders elongation and DSA-serrations, but increases the tendency of orange peel formation.
(ii) Generally, rapid cooling from increased final annealing temperatures (SB treatment) results in reduction or absence of Lüdering and consequently improved surface properties.
(iii) Reduction of Mg leads to reduced stretcher strain marks of both types, but at the expense of tensile strength.
(iv) Variation of the Mn content to the extent applied has a slightly positive effect on the surface quality in case of rapid cooling from 500 °C. The yield stress is marginally reduced.
(v) Additions of the dispersoid formers Zr and Er may have a minimal effect on the occurrence of stretcher strain marks. However, for the SB-treated sheets the Zr modification results in significantly improved mechanical properties.
(vi) Rapidly cooled modifications with Zn generate an enhanced optical surface appearance, and simultaneously improved mechanical properties. Within the first 5% of plastic deformation, the DSA serrations are also strongly suppressed.

The effect of the terminal cold-rolling degree on the Lüders strain can be explained by the CR’s influence on the final grain size. It is well known that the recrystallization of a low cold-worked structure produces large grains, and vice versa [1]. Accordingly, 20% CR alloys exhibit relatively large grains, while grains in 63% CR-processed alloys are comparatively small. Moreover, it has been known for some time that a fine-grained structure tends to pronounced Lüdering. The applicable relationship—ε_L_∝*d*^−0.5^—was recently elucidated by the authors of this study using a new metal‒physical approach [36]. While large grains result in reduced Lüders elongation, they increase the material’s proneness to the orange peel effect. Details to orange peel formation are provided below.

The reduced or completely suppressed Lüdering when SB treatment (annealing at high temperature, followed by rapid quenching) is applied may be explained by the formation of non-aged dislocations in the vicinity of coarse intermetallic particles (constituents). These are created by utilization of the difference in the thermal expansion of matrix and constituents upon quenching. The activation of pinned dislocation sources, which causes Lüdering [36,62], thus becomes obsolete. In addition it can be noted that the SB treatment produced good elongation values (e.g., Figure 3a), which we attribute to a stronger proportion of recrystallization over recovery.

Mg exhibits a distinct tendency to segregate on grain boundaries [63]. Consequently, it can be assumed that dislocation sources in or near the boundary experience weaker Mg pinning with decreasing Mg content [62]. Easier activation of dislocation sources, in turn, produces reduced Lüders elongation and Lüders lines. The reduced yield stress with lower Mg content can be explained by two things: reduced solid solution hardening and reduced grain boundary hardening due to a reduced Hall‒Petch coefficient [36].

Manganese plays a dual role in the properties described. The Mn content affects the Mn-dispersoids, which in turn influence the grain size and thus the Lüders strain according to the relation ε_L_∝*d*^−0.5^, but they also determine the extent of free dislocations by the number and size of coarse particles. There might be still coarse constituents to generate mobile dislocations at reduced Mn-content (Figure 10), but also the grain size increases. As a result of this complex situation, reduced Lüdering and yield stress were observed.

Adding Zr and Er has only a minimal effect on the occurrence of stretcher strain marks. Both elements form dispersoids of Al_3_(Zr,Er) type, which retard recrystallisation and normal grain growth due to their Zener pinning force [53,54,64,65,66]. Despite the reduced grain size in the Zr-modified alloy the visual appearance does not deteriorate. This slight positive influence on Lüdering of SB sheets may be related to that slightly increased number of mobile dislocations caused by the presence of Al_3_Zr dispersoids. The increased yield stress of the Zr-modified sheet in SB condition can be explained by reduced grain size and a partially non-recrystallized microstructure.

The almost non-existent influence of Er on recrystallization and on the surfaced quality can be related to its low content: 0.08 wt % Er correspond to 0.014 at %, which is probably too low to generate much effect. Er is known to improve the strength by grain refinement [67]. In our case, however, no significant grain refinement was achieved. The low content may also be responsible for the very weak influence on the mechanical properties.

The improved optical surface quality and the enhanced mechanical properties after Zn addition—and to a lower extent after Cu addition—is related to natural ageing (NA) effects (note that the sheets were stored at room temperature for a period of >14 days prior to tensile testing). For 5xxx-series alloys, it is known that the serrated flow is reduced when Zn is added. This effect is caused by the formation of Zn‒Mg clusters (T-phase (Mg_32_(Al, Zn)_49_) precursor) during NA, which causes depletion of solute Mg in the matrix [68,69]. A similar effect can be assumed for Cu‒Mg cluster formation in Cu-modified alloys [55]. Likewise, the marked increase in strength can be attributed to the hardening effect of the clusters/GP zones formed during NA [50].

It is important to be aware that only high temperature annealing and rapid cooling result in a favourable combination of surface appearance and mechanical properties. The annealing temperature must be chosen above the solvus of the T phase (348 °C in the case of alloy Zn2, calculated using Pandat [70]), and the alloying elements must be kept in solid solution. While with the ACF treatment the annealing temperature is, at 370 °C, just above T-solvus, slow furnace cooling generates too weak a supersaturation and the positive effect of Zn is lost. It may be very significant that Zn-modified alloys exhibit remarkable particle hardening potential, and that artificial aging can lead to a significant increase in strength [50,71]. This may mean new and wider applications for alloys of the 5xxx family.

A large number of alloy modifications in different conditions does not show pronounced Lüdering, but the appearance of a kink in the σ-ε curve, i.e., a peculiarity at the onset of plastic deformation. Interestingly, the occurrence of a kink does not necessarily generate the emergence of Lüders lines; see Table 2. In contrast to classic Lüdering, it is assumed that where there are kinks mobile dislocations are present, but not in sufficient numbers to allow for complete strain transfer across the grains. As plastic deformation progresses, however, the stress level for activation of Mg-pinned dislocation sources is reached and plastic deformation continues with ‘normal’ strain hardening. This process blurs the transition from elastic to plastic deformation and thus weakens the appearance of Lüders lines.

The well-known observation of a certain grain size threshold after which the orange peel effect occurs, 40 to 70 μm [35], does not apply to all sheets examined: the SB-processed sheets exhibit first indications of orange peel at lower grain size than those processed with ACF. The authors attribute this behaviour to the difference of the grain shape; the ACF alloys retain features of the rolling microstructure, i.e., the grains are somewhat compressed in T direction, which apparently has a reducing effect on the formation of undesired surface roughening. On the other hand, the equiaxed grains of the SB route seem to be more susceptible to orange peel formation. This finding seems reasonable considering that orange peel roughness results from grain rotations caused by the requirement of geometric compatibility with neighbouring grains [72,73,74].

## 5. Conclusions

This work investigates the influence of various alloying elements on the post-deformation surface quality and the mechanical properties of an AlMgMn alloy of EN AW-5182 type. Sheets were produced via different processing routes (63% and 20% final cold-rolling degree, soft annealing in air-circulated furnace with subsequent furnace cooling and salt-bath heat treatment with subsequent water quenching, respectively) and assessed via metallographic inspection and tensile testing. Special focus was put on the formation of Lüders lines, DSA stretcher strain marks, and orange peel formation during plastic deformation. The most important findings are summarized as follows:

- For various alloy modifications of EN AW-5182, final soft annealing at 500 °C with subsequent water quenching generates a significant reduction or total suppression of the Lüders elongation and—as a consequence—improved surface quality in terms of Lüders lines. With a low degree of cold rolling (20%), orange peel formation may occur even at a grain size of 30–40 µm.
- Modifications of the main alloying elements Mg and Mn cause no extraordinary changes in the optical and mechanical properties. Reducing the Mg content improves the surface quality, but at the expense of tensile strength. An intermediate Mn content provides a good balance between surface quality and strength, even though the effect of Mn on the surface quality is small and only manifested with rapid cooling.
- Adding Zr largely inhibits recrystallization, which is reflected in a textured microstructure. Adding Er affects neither the surface quality nor the mechanical properties.
- Specific combinations of Zn or Cu addition, cold-rolling degree, and heat treatment generate significant improvements in the mechanical and optical properties. In particular, a Zn addition of 2 wt % generates increased strength after rapid cooling and natural ageing with a later onset of DSA upon deformation. With 63% cold rolling, the surface quality is especially good.

## Figures and Tables

**Table 1:** Chemical composition of the investigated alloys in wt %; measured via optical emission spectrometry (SPECTROMAXx from SPECTRO, Kleve, Germany). Two variants each with different content in Mg, Mn, Cu and Zn; two alloys with Zr and Er, respectively.

Alloy	Mg	Mn	Fe	Si	Cu	Zn	Zr	Er	Al
Standard	4.57	0.41	0.19	0.13	–	–	–	–	Bal.
Mg1	4.16	0.42	0.20	0.14	–	–	–	–	Bal.
Mg2	3.60	0.43	0.19	0.13	–	–	–	–	Bal.
Mn1	4.89	0.34	0.20	0.13	–	–	–	–	Bal.
Mn2	4.48	0.20	0.20	0.13	–	–	–	–	Bal.
Cu1	4.62	0.42	0.18	0.13	0.15	–	–	–	Bal.
Cu2	4.69	0.31	0.30	0.14	0.75	–	––	–	Bal.
Zn1	4.53	0.40	0.18	0.13	–	0.24	–	–	Bal.
Zn2	4.57	0.42	0.19	0.13	–	2.08	–	–	Bal.
Zr	4.72	0.34	0.32	0.14	–	–	0.15	–	Bal.
Er	4.89	0.46	0.21	0.16	–	–	–	0.08 ^1^	Bal.

^1^ Er content measured via ICP-OES.

**Table 2:** Rating of the surface quality of all tested alloy, ranging from 1 (perfect surface) to 5 (worst-case surface quality); ε_L_—extent of Lüders elongation; indication of orange peel effect; mean grain size in L (longitudinal/parallel to rolling direction) and T (transversal/normal to rolling direction) direction.

Alloy	Cold-Rolling Degree (%)	Heat Treatment	ε_L_ (%)	Rating Lüders Lines	Rating Type B SSM	Orange Peel Effect	Mean Grain Size L Direction (µm)	Mean Grain Size T Direction (µm)
5182 standard	63	ACF	1.3	5	5	No	14.4	13.1
	20	ACF	–	2 *	3	No	20.7	11.9
	63	SB	0.7 **^k^**	1	3	No	15.2	15.1
	20	SB	–	2 *	2	Slightly	34.1	26.5
Mg1	63	ACF	1.2	4	4	No	15.8	16.0
	20	ACF	–	2 *	3	Yes	47.0	40.3
	63	SB	0.5 **^k^**	1	2	No	16.0	16.0
	20	SB	–	2 *	2	Slightly	35.8	39.7
Mg2	63	ACF	0.6	3	4	No	15.9	16.5
	20	ACF	–	2 *	3	Slightly	32.8	28,1
	63	SB	0.6 **^k^**	1	2	No	15.0	16.4
	20	SB	–	1 *	2	Yes	40.3	40.8
Mn1	63	ACF	1.3	5	5	No	15.2	15.4
	20	ACF	0.1 **^k^**	2 *	2	Yes	74.2	56.6
	63	SB	0.4 **^k^**	1	2	No	17.1	17.3
	20	SB	–	1 *	2	Slightly	36.8	35.6
Mn2	63	ACF	1.4	4	4	No	16.1	16.1
	20	ACF	0.1 **^k^**	2 *	2	Yes	68.1	52.7
	63	SB	0.2 **^k^**	1	2	No	26.7	27.6
	20	SB	–	1 *	2	Slightly	38.9	44.3
Zr	63	ACF	1.1	4	4	No	17.7	14.8
	20	ACF	0.2 **^k^**	2 *	2	No	18.9	21.0
	63	SB	1.0	2	3	No	11.0	11.9
	20	SB	–	1 *	3	Slightly	19.1	23.2
Er	63	ACF	1.1	3	4	No	13.3	13.3
	20	ACF	0.5	2 *	3	Yes	33.5	33.3
	63	SB	0.8	2	3	No	16.0	17.0
	20	SB	–	1 *	3	Yes	27.3	26.7
Cu1	63	ACF	1.4	4	4	No	13.7	14.5
	20	ACF	0.2 **^k^**	2 *	3	Yes	37.2	36.5
	63	SB	0.5 **^k^**	1	3	No	23.2	24.0
	20	SB	–	2 *	2	Yes	30.7	31.1
Cu2	63	ACF	0.9	4	4	No	13.0	12.1
	63	SB	0.4 **^k^**	1	3	No	21,3	20,6
Zn1	63	ACF	1.0	4	4	No	15.7	18.3
	20	ACF	0.3 **^k^**	1 *	2	Yes	38.4	39.5
	63	SB	0.5 **^k^**	1	3	No	14.3	16.5
	20	SB	–	1 *	3	Slightly	31.4	31.9
Zn2	63	ACF	0.4	2	3	No	16.0	16.1
	20	ACF	–	1 *	2	Slightly	52.6	45.6
	63	SB	0.8	2	3, 1 **^+^**	No	11.4	11.5
	20	SB	–	1 *	3, 1 **^+^**	Slightly	28.0	28.9

* Orange peel effect and/or early stages of type B stretcher strain marks may overlay Lüders elongation and influence the rating; **^k^** Discontinuity in the σ-ε curve; appearance of a kink, which is not rated as a typical Lüders elongation; **^+^** Concerns the first 5% plastic strain.

## Appendix A

**Table A1:** Sample designation and detailed sample description.

Sample Designations	Description
63% ACF	63% cold-rolling degree with soft annealing treatments in an air-circulated furnace at 370 °C for 1 h with subsequent furnace cooling
20% ACF	20% cold-rolling degree with soft annealing treatments in an air-circulated furnace at 370 °C for 1 h with subsequent furnace cooling
63% SB	63% cold-rolling degree with soft annealing treatments in salt bath at 500 °C for 5 min with subsequent water quenching
20% SB	20% cold-rolling degree with soft annealing treatments in salt bath at 500 °C for 5 min with subsequent water quenching

**Table A2:** Mechanical properties of the tested alloy variations. *R*_p0.2_—0.2% proof stress (mean standard deviation is ~2.5 MPa); *R*_m_—ultimate tensile strength (mean standard deviation is ~3 MPa); *A*_80_—elongation at failure (initial gauge length 80 mm, mean standard deviation is ~1.3%).

Alloy	Cold Rolling Degree (%)	Heat Treatment	*R*_p0.2_ (MPa)	*R*_m_ (MPa)	*A*_80_ (%)
5182 standard	63	ACF	140	275	21
	20	ACF	132	277	20
	63	SB	120	268	24
	20	SB	108	262	23
Mg1	63	ACF	125	253	21
	20	ACF	106	251	21
	63	SB	116	258	22
	20	SB	102	247	23
Mg2	63	ACF	120	245	21
	20	ACF	115	245	18
	63	SB	108	244	23
	20	SB	98	237	22
Mn1	63	ACF	137	174	21
	20	ACF	108	166	21
	63	SB	116	163	23
	20	SB	114	268	23
Mn2	63	ACF	126	256	21
	20	ACF	98	249	22
	63	SB	100	248	22
	20	SB	97	241	25
Zr	63	ACF	118	279	18
	20	ACF	103	268	17
	63	SB	195	362	24
	20	SB	183	368	20
Er	63	ACF	140	283	22
	20	ACF	113	269	23
	63	SB	123	275	25
	20	SB	111	275	22
Cu1	63	ACF	144	292	21
	20	ACF	121	292	21
	63	SB	121	280	26
	20	SB	116	288	23
Cu2	63	ACF	139	287	19
	63	SB	135	303	25
Zn1	63	ACF	133	276	21
	20	ACF	109	265	23
	63	SB	124	272	24
	20	SB	110	269	24
Zn2	63	ACF	118	279	18
	20	ACF	103	268	17
	63	SB	195	362	24
	20	SB	183	368	20
